# A Review of Recent Advances in Unidirectional Ultrasonic Guided Wave Techniques for Nondestructive Testing and Evaluation

**DOI:** 10.3390/s25041050

**Published:** 2025-02-10

**Authors:** Ali Abuassal, Lei Kang, Lucas Martinho, Alan Kubrusly, Steve Dixon, Edward Smart, Hongjie Ma, David Sanders

**Affiliations:** 1 School of Electrical and Mechanical Engineering, University of Portsmouth, Portsmouth PO1 3DJ, UK; ali.abuassal@port.ac.uk (A.A.); edward.smart@port.ac.uk (E.S.); hongjie.ma@port.ac.uk (H.M.); david.sanders@port.ac.uk (D.S.); 2 Department of Electrical Engineering, Pontifical Catholic University of Rio de Janeiro, Rio de Janeiro 38097, Brazil; lucas.martinho@cpti.cetuc.puc-rio.br (L.M.); alan@cpti.cetuc.puc-rio.br (A.K.); 3 Department of Physics, University of Warwick, Warwick CV4 7AL, UK; s.m.dixon@warwick.ac.uk

**Keywords:** ultrasonic guided waves (UGWs), unidirectional UGWs, nondestructive testing and evaluation (NDT&E)

## Abstract

Unidirectional ultrasonic guided waves (UGWs) play a crucial role in the nondestructive testing and evaluation (NDT&E) domains, offering unique advantages in detecting material defects, evaluating structural integrity, and improving the accuracy of thickness measurements. This review paper thoroughly studies the state of the art of unidirectional UGWs before presenting a comprehensive review of the foundational mathematical principles of unidirectional UGWs, focusing on the recent advancements in their methodologies and applications. This review introduces ultrasonic guided waves and their modes before looking at mode excitability and selectivity, signal excitation, and mechanisms used to generate and receive guided waves unidirectionally. This paper outlines the applications of unidirectional UGWs to reflect their effectiveness, for instance, in measuring thickness and in identifying defects such as cracks and corrosion in pipelines, etc. The paper also studies the challenges associated with unidirectional UGW generation and utilisation, such as multi-mode and side lobes. It includes a review of the literature to mitigate these challenges. Finally, this paper highlights promising future perspectives and develops directions for the technique. This review aims to create a useful resource for researchers and practitioners to comprehend unidirectional ultrasonic guided waves’ capabilities, challenges, and prospects in NDT&E applications.

## 1. Introduction

Ultrasonic guided waves (UGWs) are mechanical waves propagating along structures, such as pipes, plates, and rods, and are bound by the surfaces or boundaries along which they propagate. Their ability to travel long distances while maintaining sensitivity to defects makes them highly valuable for applications like nondestructive testing and evaluation (NDT&E), structural health monitoring (SHM), and industrial inspection. Guided waves offer advantages over bulk waves because they can propagate through complex geometries and reach areas that would otherwise be inaccessible [1,2,3]. UGWs propagate as distinct modes with dispersion relationships that depend on the material properties and waveguide geometry of the sample [4,5,6,7,8,9].

Various techniques can be used to generate guided waves, depending on the desired frequency, type of wave mode, and specific application. Source arrays, for example, generate waves of diverse natures (such as electromagnetic [10,11,12], seismic [13,14], or acoustic [15,16,17,18,19]). These applications rely on the generation of electromagnetic waves by phased arrays of antennae [10] or ultrasonic waves by piezoelectric [17,19,20,21,22,23], magnetostrictive [24,25,26,27], or electromagnetic acoustic transducers (EMATs) [15,17,28,29,30]. In structures that behave as waveguides, arrays are often arranged with their elements placed along the direction of wave propagation and driven by the same stimulus. This configuration is called a single-array transducer, also known as a comb transducer [31].

In 2024, Cawley examined the principles, historical applications, and future prospects of guided waves in long-range NDT and SHM in [32]. He concluded that using guided waves in long-range NDT is relatively advanced but primarily limited to simple structures and also that guided waves are particularly beneficial when conventional point inspection methods are inadequate or impossible due to limited access [32]. Since guided waves propagate in multiple directions, energy spreads in relatively wide directions. This can result in reduced energy concentrations, which makes defect detection over long distances more challenging due to attenuation and energy loss. Moreover, propagation in multiple directions increases the reflections from boundaries, obstacles, and defects, which can cause interference and reduce the clarity of the signal. This complicates data interpretation and can mask minor defects. One can focus the wave energy by constraining the wave propagation into a single direction, also known as “*unidirectional guided waves*” [23,33,34], which increases the efficiency of wave propagation and improves the detection range while reducing energy loss due to less scattering in other directions. Furthermore, there is a reduced need for compensation techniques to enhance the signal, such as signal amplification or noise reduction algorithms. Also, reflections from boundaries or defects in other directions are minimised, resulting in better signal clarity and reduced noise. This is particularly important for defect detection in complex geometries or materials with high interference levels [35,36].

Unidirectional guided waves are a class of elastic waves that propagate in a single direction within a bounded medium, such as a plate [37], or a pipe [34]. The geometry of the medium guides [38] these waves, containing their energy and directing their path. They can be utilised when guided waves are used but work more efficiently and accurately [39,40]. Unidirectional guided waves offer significant advantages in various fields, including NDT&E and SHM. Their directed energy and wave propagation make them ideal for applications requiring selective wave modes and high-resolution detection. While challenges such as mode conversion, side lobes, and environmental interference remain ongoing, research and technological advances are addressing these issues. With the continued development of transducer technology, unidirectional guided waves are poised to play an increasingly vital role in future industrial and research applications [41,42]. Recently, some unidirectional transducers have been developed to generate shear horizontal (SH) [27,43,44], Lamb [45,46,47], shear vertical [48,49], torsional [38,39], longitudinal [34,38], and Rayleigh waves [17,50] and have enhanced the wave energy propagated along one desired direction. Most unidirectional transducers are formed by two bidirectional transducers with phase- or time-delay control between them [48].

This paper is organised as follows: First, we introduce the UGW modes in Section 2, a fundamental concept that underpins the rest of the discussion. Then, we describe the principle of generating and receiving unidirectional ultrasonic guided waves in Section 3, before demonstrating how to generate optimal unidirectional UGWs and tackle the challenges related to their generation in Section 4. In Section 5, we outline the ultrasonic transducers that generate ultrasonic waves unidirectionally. Finally, we show examples of unidirectional UGW applications in Section 6, before concluding with a prediction of future directions.

## 2. Ultrasonic Guided Wave Modes

Each mode of ultrasonic guided waves, such as SH, Lamb, Rayleigh, etc., offers distinct advantages and challenges, making them suitable for different applications [51,52]. Using a carefully designed ultrasonic transducer, one can excite and select a specific UGW mode, mitigating ultrasonic reflections from non-preferred directions and interference from undesirable modes. Different modes can be excited depending on the driving signals’ characteristics, ultrasonic transducer type, and the geometric properties of the structure [42,51,53]. Due to dispersion, the frequency components of each mode can be distributed widely in the time domain, making it challenging to separate individual modes [51,54,55,56,57].

### 2.1. Rayleigh Waves

Ultrasonic Rayleigh waves propagate along the surface of an elastic solid, with their amplitude decaying exponentially with depth. They are used extensively in NDT&E to detect surface and near-surface defects in materials [52,58]. In a medium, the particle motion associated with the Rayleigh wave is elliptical, with the ellipse’s central axis perpendicular to the surface and its minor axis parallel to the direction of wave propagation. The motion is retrograde (opposite to the direction of wave propagation) at the surface [51,59,60,61].

For a semi-infinite solid surface, the displacement field *u* is governed by the equations of motion derived from the Navier–Cauchy equations [62]:

(1) $$\mu {▽}^{2}u+\left(℘+\mu \right)▽\left(▽.u\right)=\rho \frac{\partial^{2}u}{\partial t^{2}}\;,$$

where

- ρ is the material density;
- *℘* and μ are the Lame parameters;
- $u=(u_{x},u_{z})$ is the displacement vector in *x* and *z*axes, and there is no displacement in the y−axis, when considering *z* as the direction normal to the surface and *x* the propagation direction.

The wave solutions are

(2) $$u_{x}\left(x,z,t\right)=U\left(z\right)e^{j(\omega t-kx)}\;,$$

(3) $$u_{z}\left(x,z,t\right)=W\left(z\right)e^{j(\omega t-kx)}\;,$$

where *k* is the wavenumber of the mode $\left(k=\omega /c_{p,R}\right)$, ω is the angular frequency, $c_{p,R}$ is the phase velocity of the Rayleigh wave, and U(z) and W(z) describe the depth dependence of the displacements in *x* and *y* axes. For the Rayleigh waves, one must satisfy the boundary conditions at the free surface (z=0), where the normal stress, $\sigma_{zz}$, and the shear stress, $\sigma_{xz}$, vanish. These conditions lead to the following characteristic equation or dispersion relation:

(4) $$\begin{array}{c} \left(\frac{2-\eta^{2}}{\sqrt{1-\eta^{2}}}-\sqrt{1-\eta^{2}}\right).\left(\frac{2-\eta^{2}}{\sqrt{1-\eta^{2}}}+\sqrt{1-\eta^{2}}\right)=0\;, \end{array}$$

where $\eta =\frac{\omega }{kc_{T}}$ and $c_{T}$ is the shear bulk wave velocity ($c_{T}=\sqrt{\mu /\rho }$, where μ is the shear modulus). One can solve the dispersion relation for Equation (4) numerically to find the phase velocity of the Rayleigh wave $c_{p,R}=\frac{\omega }{k}$ as a function of frequency and the wavenumber *k*. An approximated solution, given by Viktorov [63] and based on Poisson’s ratio, for the Rayleigh wave is given by

(5) $$\frac{c_{p,R}}{c_{T}}\approx \frac{0.87+1.12\nu }{1+\nu }\;,$$

where ν is the Poisson’s ratio.

### 2.2. Shear Horizontal (SH) Waves

SH waves propagate through a medium with particle motion entirely in the horizontal plane, perpendicular to the direction of wave propagation. Unlike other guided waves, such as Rayleigh and Lamb waves, which involve both in-plane and out-of-plane motion, SH waves are purely in-plane waves, meaning they do not have a vertical displacement component. SH waves are particularly useful in detecting defects affecting surface or near-surface regions [51,64]. The fundamental mode (SH0) is the only non-dispersive SH mode in isotropic, homogeneous plates [44,65].

**Figure 1:**
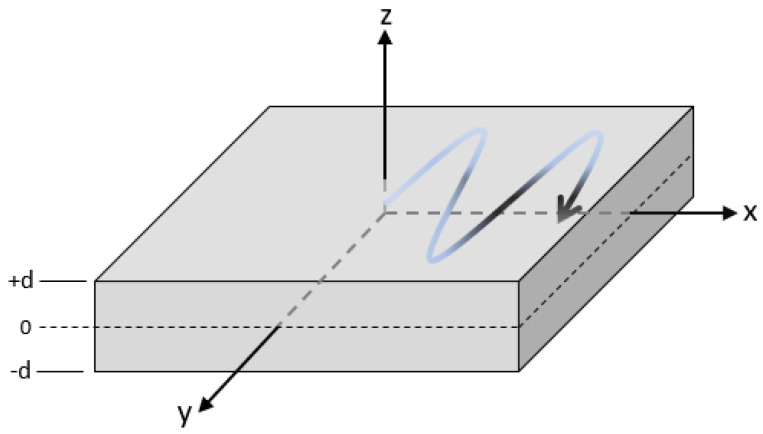
Propagation of SH wave mode, illustrated by the gradient arrow, in a plate with a thickness of 2d, where the propagation is along *x* and the material particle displacements are along *y*.

Consider the aluminium plate in Figure 1, which has a thickness 2d, where an SH wave mode propagates along the *x* direction, with particle displacements in the *y* direction [66,67]. The wave equation (Equation (6)) governing SH waves propagating in an elastic medium along the *x*-axis with displacement $u_{y}\left(z\right)$ in the *y* direction (perpendicular to the *x*-axis and within the plane of the plate) is

(6) $$\frac{\partial^{2}u_{y}}{\partial t^{2}}={c_{T}}^{2}\left(\frac{\partial^{2}u_{y}}{\partial x^{2}}+\frac{\partial^{2}u_{y}}{\partial z^{2}}\right).$$

Similar to the Rayleigh wave case, the *y* displacement component of the SH UGW can be written as

(7) $$u_{y}\left(x,z,t\right)=V\left(z\right)\;e^{j(\omega t-kx)}\;.$$

Substituting Equation (7) into Equation (6) yields

(8) $$\frac{\partial^{2}u}{\partial z^{2}}+\left(\frac{\omega^{2}}{c_{T}^{2}}-k^{2}\right)V\left(z\right)=0\;.$$

Considering the boundary conditions of free stress at the plate’s surface and solving for the phase velocity of the SH wave, $c_{p,SH}$, the solutions to Equation (8) can be written as

(9) $$c_{p,SH}\left(fd\right)=2c_{T}\left(\frac{2fd}{\sqrt{4{\left(2fd\right)}^{2}-n^{2}c_{T}^{2}}}\right)\;.$$

**Figure 2:**
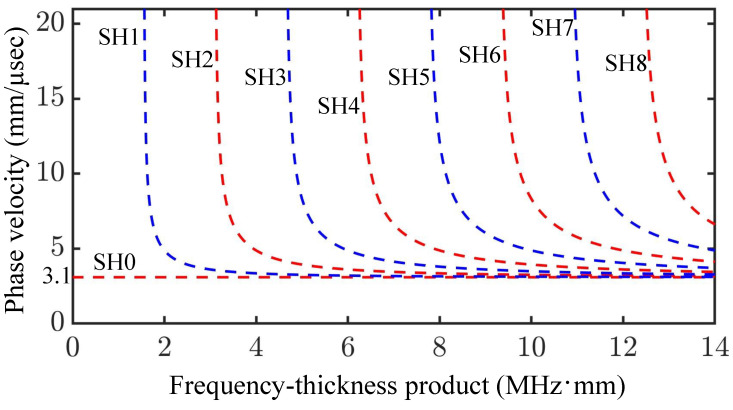
SH-mode phase velocity dispersion curves for a flat plate of aluminium with a 5 mm thickness and bulk shear wave velocity of $c_{T}$ = 3.1 mm/μs over a frequency–thickness product range of 0–14 MHz·mm. SH0 represents the fundamental mode, which is the only non-dispersive mode and has the same phase velocity as the bulk shear wave. The other modes, SH1 to SH8, are dispersive modes. These dispersion curves were generated using Dispersion Calculator software (v3.0) [68].

Equation (9) details the dispersive relationship of SH UGWs in a flat plate and is plotted in Figure 2, which shows the phase velocity dispersion curves for the first eight SH UGW modes over a frequency–thickness product range of 0–14 MHz·mm with a bulk shear wave velocity of 3.1 mm/μs. The SH0 mode (zeroth-order SH mode, or fundamental mode), is a non-dispersive wave, propagating at the shear wave velocity (shown as a straight line at 3 mm/μs). All other SH UGW modes are dispersive.

### 2.3. Lamb Waves

Ultrasonic Lamb waves propagate as multiple dispersive UGW modes in thin plates or layered structures. Referring to Figure 1, Lamb waves have particle displacements in the *z* and *x* directions. Lamb wave and Lamb-like UGW modes are utilised in NDT&E, SHM, and material characterisation [51,69,70,71]. As with SH modes, Lamb modes are symmetric (S) or anti-symmetric (A), relative to the middle plane of the plate [47,51,72].

Lamb waves can propagate over long distances along plates and other structures, making them highly useful for complex structures where it is essential for a transducer to assess a large area of the surrounding structure. However, there are various modes of Lamb waves, and to obtain clear signals that can be reliably interpreted, it is crucial to excite a single mode in a well-controlled direction [73]. Wilcox et al. [73] have outlined a systematic approach to selecting appropriate Lamb wave modes and operating frequencies tailored to specific inspection tasks.

Consider the plate shown in Figure 1, of thickness 2d with a longitudinal bulk wave velocity $c_{L}$, shear bulk wave velocity $c_{T}$, and density ρ. The dispersion relation is found by solving the Rayleigh–Lamb frequency equations, yielding solutions for the symmetric and anti-symmetric modes [51,74].

For symmetric modes,

(10) $$\frac{tan(qd)}{tan(pd)}=-\frac{4k^{2}pq}{{\left(q^{2}-k^{2}\right)}^{2}}\;.$$

For anti-symmetric modes,

(11) $$\frac{tan(qd)}{tan(pd)}=-\frac{{\left(q^{2}-k^{2}\right)}^{2}}{4k^{2}pq}\;.$$

Here, *p* and *q* are given by $p^{2}={\left(\omega /c_{L}\right)}^{2}-k^{2}$ and $q^{2}={\left(\omega /c_{T}\right)}^{2}-k^{2}$.

**Figure 3:**
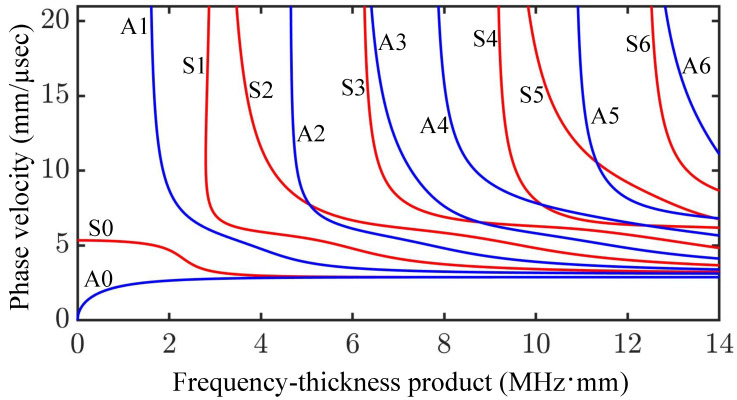
Lamb wave dispersion curves for symmetric and anti-symmetric modes in an aluminium plate of a 5 mm thickness with a bulk shear wave velocity of $c_{T}$ = 3.1 mm/μs over a frequency–thickness product range of 0–14 MHz·mm. The red lines represent the symmetric modes S0 to S6, and the blue lines represent the anti-symmetric modes A0 to A6. Modes A0 and S0 are the fundamental modes. This dispersion curve was generated using Dispersion Calculator software (v3.0) [68].

Since the Rayleigh–Lamb equations are transcendental, they are solved by numerical methods. Figure 3 shows the phase velocity dispersion curves for the symmetric and anti-symmetric modes over a frequency–thickness product range of 0–14 MHz·mm and bulk shear wave velocity of $c_{T}$ = 3.1 mm/μs [51,75].

In addition to the SH and Lamb UGW modes, there are many other guided wave modes, such as torsional [76,77,78,79,80], longitudinal [81,82,83], flexural [84,85,86,87], Love [88,89,90], helical waves [91,92,93], and Lamb-like modes, that have been widely utilised for the inspection of pipes [76,81,85,94,95], rails [48,96], cables [83,97], and aerospace structures [98].

While guided waves can provide significant advantages for long-range and efficient inspection, they usually propagate in omnidirectional or bidirectional directions. As a result, their successful implementation requires one to address several challenges, such as signal interpretation complexity, geometrical energy loss, and the risk of “false alarms” [51,99]. In a component or structure, UGW modes can propagate and scatter in undesirable directions. Unidirectional ultrasonic guided waves, on the other hand, can help to solve some of these challenges by reducing the system’s complexity and potential for “false alarms” by ensuring that waves are received mainly from one desired direction and that waves originating from other unwanted directions are not generated in the first place or are removed from the received signal [100,101].

## 3. Principle of the Generation and Reception of Unidirectional UGWs

The conventional transducer for UGWs usually generates waves in both forward and backward directions. In contrast, the principle for generating and receiving unidirectional UGWs commonly involves using arrays of UGW transducers, carefully designed signal excitations, and interpreting techniques to ensure that waves propagate in a single direction while also being able to detect or receive them efficiently. Unidirectional guided waves offer the advantages of a better signal-to-noise ratio and focused wave energy, which are critical for some applications [33,38,100].

### 3.1. Principle of Unidirectional UGW Generation

The principle of generating unidirectional UGWs consists of generating and directing ultrasonic guided waves into a controlled direction along a structure or medium. The unidirectional propagation of guided waves can be achieved through various mechanisms and design strategies [33,69,102,103].

**Figure 4:**
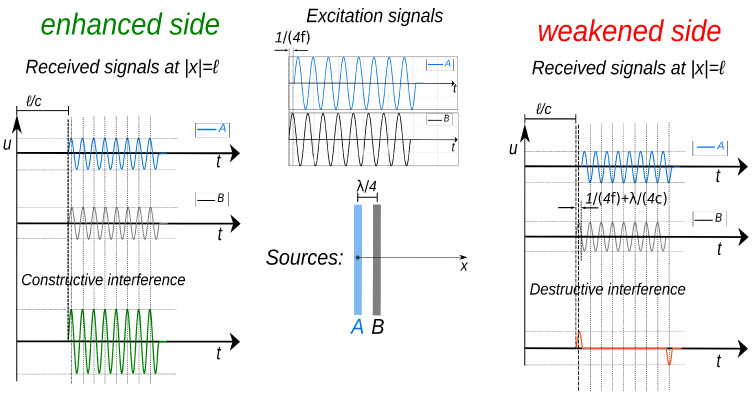
This diagram illustrates a general example to explain the principle of generating unidirectional ultrasonic guided waves. The blue and grey rectangles represent sources A and B, which simultaneously emit ultrasonic waves in both forward and backward directions. The excitation signals ((**top middle**) graph) are depicted with different colours corresponding to their sources. The weakened side is shown on the right-hand side, with the individual and total waves resulting from the controlled destructive interference. Conversely, the left-hand side demonstrates the enhanced side, where the controlled constructive interference occurs. Redrawn based on [104].

A typical method for controlling the direction of ultrasonic guided waves is to employ two identical bidirectional wave sources separated by a quarter of the wavelength of the generated waves. These two transducers are triggered one after the other by a delay of a quarter-period of the signal time. This way, constructive interference occurs in one direction, “*the enhanced side”*, whilst destructive interference appears in the opposite direction, “*the weakened side*”, making the entire wave source unidirectional [33,103]. This approach is illustrated in Figure 4, using two equivalent wave sources (A and B), which are separated by a predefined distance, Δx, and can be excited independently, following [104]. Assuming a monochromatic signal at the angular frequency, 2πf, excited from both sources with a time delay of Δt, the generated ultrasonic wave from each ultrasonic source along the *x*-axis is given by

(12) $$u_{A}=e^{j[\omega t\pm kx]}\;,$$

(13) $$u_{B}=e^{j[\omega (t+\Delta t)\pm k(x+\Delta x)]}\;,$$

where λ is the wavelength, and the symbol ± explains the wave’s propagation to the left side (the negative direction on the *x*-axis) and right side (the positive direction on the *x*-axis), respectively. The typical propagation of waves from each ultrasonic transducer is seen in Figure 4.

Generally, Equations (12) and (13) are considered unit amplitude waves. The unidirectional generation of ultrasonic guided waves can be achieved by establishing Δt and Δx precisely; the time delay is a quarter of a period, i.e., Δt=π/2ω; and the distance between the sources is a quarter of a wavelength, i.e., Δx=π/2k. Then, Equations (14) and (15) represent the resulting total wave propagation on the left and right sides, respectively [104].

(14) $$u_{l}=e^{j[\omega t-kx]}+e^{j[\omega (t+\pi /2\omega )-k(x+\pi /2k)]}=2e^{j[\omega t-kx]}\;,$$

(15) $$u_{r}=e^{j[\omega t+kx]}+e^{j[\omega (t+\pi /2\omega )+k(x+\pi /2k)]}=0\;,$$

where $u_{l}$ and $u_{r}$ are the total wave propagation on the left and right sides of the ultrasonic sources, respectively.

The aforementioned example is considered a single-frequency signal. However, this principle still holds for finite-length pulses [104], as schematically shown in Figure 4, where the two rectangles coloured blue and grey represent the two separate ultrasonic sources, A and B, which are polarised to generate the required wave mode. Source B is moved to the right, causing its waves to come earlier than those generated by A for positions on the right side and later on the opposing side. However, the time delay set at A makes both waves arrive in-phase on the left-hand side and out-of-phase on the right-hand side, delivering enhanced and weakened waves on the left- and right-hand sides, respectively. Ideally, this concept works for any unidirectional UGW mode, even though the authors in [104,105] utilised this principle to generate shear horizontal waves, provided that the appropriate distance between the sources is introduced along the direction of propagation. However, destructive and constructive interference only emerge at the frequency for which the separation equals a quarter-wavelength. That means the relation f=c/λ must be considered; otherwise, the phase delay does not match the separation, and ideal wave suppression does not occur.

Furthermore, it is still valid for the separation between the two sources to be any odd multiple of λ/4, as long as the time delay matches. Nevertheless, most minor applicable separations inherently mitigate any pulse-changing phenomena, such as dispersion and attenuation [103,106,107,108]. Moreover, applying the same principle to pipelines, one can utilise two rings of transducers to generate unidirectional UGWs, such as torsional and longitudinal guided waves, as in [34,39].

The time delay must precisely match the spacing of the related wave source to satisfy the interference principle. As a result of strictly following the delay of a quarter-period of the signal time, this principle can only work for a narrow range of frequencies. However, a wide range of frequencies is crucial whenever a wide bandwidth is required for an application. Transducers that generate unidirectional UGWs over a wide range of frequencies have been proposed, such as a time-delayed layered piezoelectric transducer [108,109], wideband SH guided wave phased-array magnetostrictive patch transducer [27], and an arrayed-coil EMAT for a spike-like pulse [110].

To generate unidirectional UGWs, one must excite the ultrasonic sources with appropriate signals, allowing them to generate the required waves. Several methods have been proposed in the literature to drive excitation signals appropriately [111,112,113]. Considering Figure 4, one can apply an excited signal to source B and another signal with a delay of a quarter-time period (90^∘^) to source A to achieve constructive and destructive interference. This method is referred to as “*time-delayed (TD) excitation*”, and the excited signals can be represented by Equation (16):

(16a) $$\begin{array}{c} \;g_{1}\left(t\right)=g_{0}\left(t-T/4\right)\;, \end{array}$$

(16b) $$\begin{array}{c} \;g_{2}\left(t\right)=g_{0}\left(t\right)\;, \end{array}$$

where $g_{0}\left(t\right)$ is a base pulse, and $g_{1}\left(t\right)$ and $g_{2}\left(t\right)$ are the excitation signals for ultrasonic sources A and B, respectively. This principle produces an ideal constructive interference for the forward-propagating waves, whilst the backward-propagating waves do not entirely coincide at the waves’ beginning and end, creating low-amplitude spike waves, as shown in Figure 4, on the weakened side [22,23,114,115].

On the other hand, one can excite a pair of signals by applying a quarter-time period of 90^∘^ to source B instead of source A and then reversing it or multiplying by −1. This method is referred to as “*time-delayed and inverse (TDI) excitation*”, and Equation (17a,b) illustrate the excitation signals generated using the TDI method.

(17a) $$\begin{array}{c} \;g_{1}\left(t\right)=g_{0}\left(t\right)\;, \end{array}$$

(17b) $$\begin{array}{c} \;g_{2}\left(t\right)=-g_{0}\left(t-T/4\right)\;. \end{array}$$

Note that each pair of generated waves from both sources arrives simultaneously at the positive side of the *x*-axis, but they are inverted and perfectly cancel each other. In this case, the individual waves from related sources are in-phase but are not perfectly aligned on the negative side, producing a total signal that is slightly modified [23,116]. In addition to the TD and TDI methods mentioned above, which introduce an electronic delay, one can introduce a mechanical delay by adding a time-delay layer, which has also been reported to achieve a similar effect as the time-delay-based method [108].

The potential of these time-based excitation methods ensures either the ideal constructive or destructive interference of the ultrasonic waves produced by the two ultrasonic arrays of a unidirectional ultrasonic transducer. This principle is based on using the time-delayed excitation method on one of the two arrays, where the delay matches the time it takes for the wave to propagate from one array to the other. Time-based excitation methods can achieve ideal constructive and destructive interference for a monochromatic signal, which is a continuous infinite signal with a constant amplitude and a single frequency. However, ideal interference can never be simultaneously achieved on both sides for a tone burst signal with a relatively narrow bandwidth and a pulse signal with a relatively broad frequency spectrum. Moreover, time-delayed methods only provide ideal constructive or destructive interference for non-dispersive wave modes [104,110]. Therefore, other excitation methods for dispersive guided waves will be exhibited in Section 4.3 for the optimal generation of unidirectional UGWs.

### 3.2. Principle of the Unidirectional Reception of UGWs

Unidirectional guided waves are designed to propagate in one specific direction, reducing interference from other directions and simplifying signal analysis. Receiving these waves involves capturing the propagated waves in order to interpret the structural condition of the material. While generating these waves has attracted considerable attention, unidirectionally receiving ultrasonic guided waves is equally essential. The accurate detection and interpretation of these waves are critical for identifying structural defects and integrities [45,115].

Waves received unidirectionally are usually initially generated unidirectionally on a single side, as mentioned in Section 3.1. Likewise, by using time-shifting and adding operations, one can post-process the generated waves with a dual array transducer, which receives waves that arrive mainly from the right- or left-hand sides. Following [115], assuming a similarity between the receiving and transmitting transducers, the received signal y(t) that holds the waves reaching the right-hand side is obtained by Equation (18) [41,108].

(18) $$y\left(t\right)=y_{A}\left(t\right)+y_{B}\left(t-T/4\right)\;,$$

where $y_{A}\left(t\right)$ and $y_{B}\left(t\right)$ are the signals received at the first and the second receivers, respectively. Meanwhile, signals arriving on the left side can be represented by Equation (19).

(19) $$y\left(t\right)=y_{A}\left(t-T/4\right)+y_{B}\left(t\right)\;.$$

In addition to applying a quarter-period time delay, other strategies are also employed to receive guided waves unidirectionally, such as time-delayed layers [27,108,109,110].

For unidirectional guided waves travelling along an enclosed path, such as the circumference of a pipe, with defects or discontinuities, the generated guided waves travelling unidirectionally inevitably arrive at the receiver from both clockwise and counter-clockwise directions. The unidirectional wave may be transmitted through the defects in one direction and reflected by the defects in the other. Under this condition, a unidirectional reception of the guided waves can be highly beneficial [22,102,116].

## 4. Optimal Generation of Unidirectional UGWs

Generating unidirectional ultrasonic guided waves requires maximising the ultrasonic energy of a desired wave mode in one desired direction and minimising it in other directions. This process is particularly challenging for ultrasonic guided waves due to their characteristics: they may be multi-modal, dispersive, and have side-lobe effects. Consequently, for the optimal generation of unidirectional UGWs with minimum side lobes, one must extensively study the mode excitability, optimal driving signals, sound source distribution (mainly determined by the shape of the ultrasonic transducers), and the modelling of the transducers.

### 4.1. Multi-Mode and Dispersion Phenomena in the Propagation of Unidirectional UGWs

The multi-modal and dispersive nature of UGWs must be considered carefully when applying them to NDT&E and SHM. Understanding how multiple modes of guided waves propagate and disperse in a medium is critical for accurately generating desired wave mode(s), correctly interpreting wave signals, and diagnosing material properties or defects [69,117]. Several elements impact the behaviour of unidirectional guided waves, including the medium’s material properties, the geometry of the structure, and the operating frequency of the guided wave(s) [102].

Multiple modes can propagate simultaneously, each with distinct characteristics, leading to superposition between wave modes and coherent noise, which cannot be removed by averaging [38]. Multi-mode phenomena refer to several modes of UGWs coexisting and propagating within the same medium, often with different phase velocities, group velocities, and frequency-dependent behaviours; therefore, a pure mode excitation is required to circumvent these [118,119].

Dispersion curves describe the propagation of guided waves, and an appreciation of them is essential for understanding the behaviour of different wave modes at various frequencies and selecting appropriate frequencies for the required application [103]. Moreover, the information obtained from the dispersion curves determines the transducers’ design [120]. In practice, the first step is to know the dispersion curves and conduct actual experiments [66].

In dispersion curves such as those in Figure 2 and Figure 3, the positive forward-travelling wave represents the phase velocity $c_{p}$ of the positive frequency–wavenumber ratio, while the backward wave represents the negative frequency–wavenumber ratio. A linear frequency–wavenumber ratio represents non-dispersive waves (constant phase velocity), whereas a non-linear relation represents a dispersive wave mode; an understanding of the dispersion and displacement characteristics of UGWs enables one to design a suitable excitation signal and predict wave excitability [51].

### 4.2. Mode Selectivity and Operating Region of Unidirectional UGWs

As established in the previous section (Section 4.1), UGWs exhibit a multi-modal nature, which complicates the post-processing of wave signals and can result in false alarms. In plates and pipes, UGWs can have as many as 50 modes below 100 kHz [38]. To ensure that the signals can be reliably interpreted, it is crucial to excite only one of these modes. Selecting the appropriate mode, driving signal, and ultrasonic source design are essential factors to operating with a pure mode [73,121,122]. The latter two factors define the operating region, which will be elaborated on later in this section.

The wave mode must be effectively and purely excited based on its sensitivity to specific types and locations of defects. For example, the Rayleigh wave mode is highly sensitive to surface defects that are oriented with their plane perpendicular to the direction of wave propagation. However, it is largely insensitive to defects located deeper than approximately one wavelength below the surface, as noted by Cawley [32]. In contrast, the zero-order symmetric Lamb wave mode (S0) behaves like an extensional wave at low frequencies, and it is sensitive to cracks that are normal to the direction of wave propagation, regardless of their depth within the thickness of the plate. The SH0 mode exhibits similar sensitivity. However, it is important to note that the S0 mode is generally insensitive to cracks whose plane runs parallel to the direction of wave propagation [32]. Furthermore, the torsional mode is sensitive to longitudinal cracks in the pipe, while longitudinal modes are insensitive to thin defects parallel to the pipe’s axis [38].

The mode tuning process is often performed empirically by exciting the transducer with a combination of windowed tone burst signals of various centre frequencies and cycles and then selecting the one that generates the best mode purity with the time-domain pulses of the shortest duration [123]. The concept of an operating region is useful for designing suitable excitation signals, and it can be extended to unidirectional UGWs, as proposed in [40]. Assume a transducer dictates a spatial wave source, $h_{0}\left(x\right)$, distributed along the *x*-axis. This could be the distribution of induced forces from an EMAT [69] or a piezoelectric transducer [31], polarised to generate the required wave mode of interest. Allowing $H_{0}\left(k\right)$ to be the Fourier transform of $h_{0}\left(x\right)$, one can write

(20) $$H_{0}\left(k\right)=F\left\{h_{0}\left(x\right)\right\}\;,$$

where *k* is the wavenumber or the spatial angular frequency, and the equation is in the spatial spectrum or wavenumber domain. Since $h_{0}\left(x\right)$ is centred at the origin, and to keep the principle of two wave sources distanced by λ/4, following [40], each source is shifted by λ/8 to the left and the right. onsequently, $h_{1}\left(x\right)=h_{0}\left(x+\lambda /8\right)$ and $h_{1}\left(x\right)=h_{0}\left(x-\lambda /8\right)$. Thus, Equation (20) can be rewritten for the two sources as follows:

(21a) $$\begin{array}{cc} H_{1}\left(k\right) & =H_{0}\left(k\right)e^{-jk\lambda /8}\;, \end{array}$$

(21b) $$\begin{array}{cc} H_{2}\left(k\right) & =H_{0}\left(k\right)e^{+jk\lambda /8}\;. \end{array}$$

Similarly, the current or voltage injected into each source determines the transducer’s temporal dependence. The temporal spectrum is given by its Fourier transform:

(22a) $$\begin{array}{cc} G_{1}\left(\omega \right) & =F\{g_{1}\left(t\right)\}\;, \end{array}$$

(22b) $$\begin{array}{cc} G_{2}\left(\omega \right) & =F\{g_{2}\left(t\right)\}\;. \end{array}$$

The operating region, where waves are predominantly excited, is the frequency locus coupled with the wavenumber [65,124]. It is defined by the Cartesian product of its temporal and spatial spectra and the sum of the operating regions of each transducer, as shown in Equation (23) [40].

(23) $$S\left(\omega ,k\right)=G_{1}\left(\omega \right)H_{1}\left(k\right)+G_{2}\left(\omega \right)H_{2}\left(k\right)\;.$$

Using Equation (21a,b), Equation (23) then becomes

(24) $$S\left(\omega ,k\right)=H_{0}\left\{G_{1}\left(\omega \right)e^{-jk\lambda /8}+G_{2}\left(\omega \right)e^{+jk\lambda /8}\right\}\;.$$

Considering Equation (24), a positive value of *k* conveys forward-propagating waves, whilst a negative value conveys a backward-propagating wave. The absolute value of the operating region S(ω,k) at the ω−k values on the wave dispersion curve controls the excitability of the selected wave mode in the desired direction, either forward or backward [65,125].

**Figure 5:**
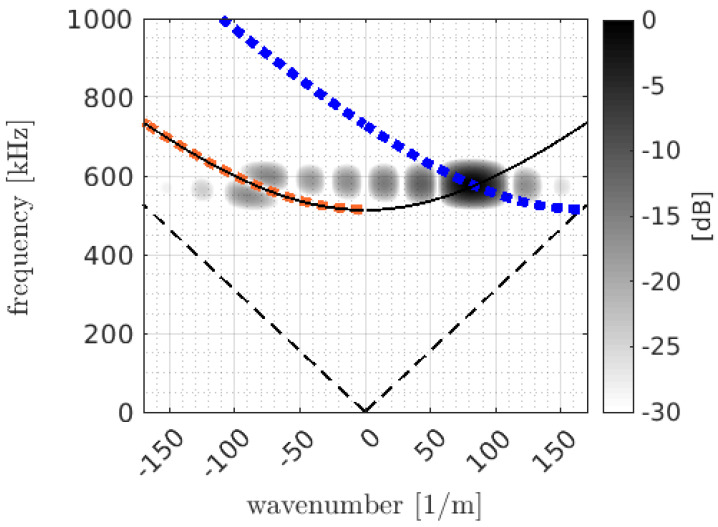
An example of an operating region for a unidirectional ultrasonic transducer that generates an SH1 mode with the base signal at a centre frequency of 577.7 kHz. The blue and orange curves demonstrate the maximum and minimum loci of interference on the positive and negative sides, respectively. The black dashed line shows the dispersion curve of the SH fundamental mode (SH0), and the black arced line is the dispersion curve of the SH1 mode. As the minimum locus aligns with the dispersive curve, ideal destructive interference can be realised. Redrawn based on [103].

The operating region of unidirectional ultrasonic guided waves refers to the specific conditions under which the guided waves propagate efficiently and with maximum excitability in a particular direction. This region is determined by factors such as frequency, wavelength, material properties, and wave mode. The principle behind the operating region involves optimising these factors to generate UGWs with high selectivity and directionality. For instance, Figure 5 illustrates the operating region (the shaded areas) of an ultrasonic transducer. The black arced line in the figure represents the dispersion curve of the SH1 waves. When the operating region aligns with the dispersion curve, SH1-mode ultrasonic guided waves will be generated. This generation process can be optimised by the careful design of the driving signals, which will be elaborated on in Section 4.3.

### 4.3. Signal Excitation for the Optimal Generation of Unidirectional UGWs

To achieve pure mode excitation, it is necessary to control at least two parameters, since two modes can exist at every frequency [32]. The most commonly controlled parameters are the frequency and wavelength of the excitation. The input excitation signal regulates the frequency, while the geometry of the transducer determines the wavelength [32]. For the input signal, one must ensure the excitation methods are suitable for the type of excitation signal used to drive the transducer’s array and the nature of the guided waves, which are highly dispersive. The time-based excitation methods (TD and TDI) mentioned in Section 3 are unsuitable for unidirectional dispersive guided waves (present in higher-order modes). Other methods proposed by [103] based on synthetic propagation provide suitable excitation methods for high-order unidirectional UGWs. These are summarised below.

#### 4.3.1. Synthetic Propagated (SP) Excitation

Using the same signal design methodology, one can meticulously apply a signal equal to the wave that arrives at one source after departing from the other, achieving optimal constructive and destructive interference. However, the propagation of a dispersive wave, with its unique phase velocity for each frequency component, does not behave as a simple time shift [103]. Therefore, we must consider the propagating operator in the frequency domain, $e^{jk_{n}\left(\omega \right)l}$ [126,127], where *l* is the propagating distance and $k_{n}\left(\omega \right)$ is the frequency-dependent wavenumber expression for wave *n*, with *n* representing the wavenumber that determines the desired wave mode(s).

Accordingly, by exciting the second wave source with a signal similar to the wave that reaches the source’s position after leaving the first wave source, theoretically, one can perform ideal constructive interference [103], which can be achieved based on the following excitation signals.

In the time domain,

(25a) $$\begin{array}{c} \;g_{1}\left(t\right)=g_{0}\left(t\right)\;, \end{array}$$

(25b) $$\begin{array}{c} g_{2}\left(t\right)=F^{-1}\left\{e^{-jk_{n}\left(\omega \right)\lambda /4}G_{0}\left(\omega \right)\right\}\;. \end{array}$$

In the frequency domain,

(26a) $$\begin{array}{c} \;G_{1}\left(\omega \right)=G_{0}\left(\omega \right)\;, \end{array}$$

(26b) $$\begin{array}{c} G_{2}\left(\omega \right)=e^{-jk_{n}\left(\omega \right)\lambda /4}G_{0}\left(\omega \right)\;. \end{array}$$

Equation (25b) and Equation (26b) represent the signal propagated from the first to the second source in the time and frequency domains, respectively, and the distance between the two sources is a quarter of the wavelength l=λ/4. Theoretically, no closed-form equation exists for the time-domain signal $g_{2}\left(t\right)$. Therefore, it must be calculated by an inverse Fourier transform ($F^{-1}$) of its spectrum, obtained from phase shifting ($G_{0}\left(\omega \right)$). For synthetic propagated excitation, one can calculate the operating region using Equation (27), which is based on Equations (24) and (26). This principle was originally devised for SH guided waves [103]. However, using the same principle, one can define various operating regions to generate different unidirectional UGW modes, such as Rayleigh, Lamb, longitudinal, Love, and torsional waves. Considering Equation (27), maximum and minimum loci appear at $k=k_{n}\left(\omega \right)$ and $k=k_{n}\left(\omega \right)-4\pi /\lambda$. This means the ideal constructive interface is acquired for the wave mode *n*, regardless of its dispersive nature. Moreover, if the mode is non-dispersive, then $k_{n}\left(\omega \right)=\omega T/\lambda$, meaning the propagating operator fits the time shift.

(27) $$S\left(\omega ,k\right)=2G_{0}\left(\omega \right)H_{0}\left(k\right)e^{\left(-jk_{n}\left(\omega \right)\lambda /8\right)}\times cos\left(\frac{k_{n}\left(\omega \right)\lambda -k\lambda }{8}\right)\;.$$

#### 4.3.2. Synthetic Propagated and Inverse (SPI) Excitation

This type of excitation is based on the time delay and inverse excitation method and is in contrast to synthetic propagated excitation [103]. That is, one can excite the first array with a synthetically propagated signal from the second array multiplied by −1 to ensure the destructive interference of a dispersive wave [103]. Then the excitation signals can be calculated using Equations (28) and (29).

We define the excitation signal using the inverse Fourier transform,

(28a) $$\begin{array}{c} g_{1}\left(t\right)=F^{-1}\left\{e^{-jk_{n}\left(\omega \right)\lambda /4}G_{0}\left(\omega \right)\right\}\;, \end{array}$$

(28b) $$\begin{array}{c} \;g_{2}\left(t\right)=g_{0}\left(t\right)\;. \end{array}$$

in the frequency domain:

(29a) $$\begin{array}{c} G_{1}\left(\omega \right)=e^{-jk_{n}\left(\omega \right)\lambda /4}G_{0}\left(\omega \right)\;, \end{array}$$

(29b) $$\begin{array}{c} \;G_{2}\left(\omega \right)=G_{0}\left(\omega \right)\;. \end{array}$$

Consequently, the operating region can be defined using Equation (30):

(30) $$S\left(\omega ,k\right)=2G_{0}\left(\omega \right)H_{0}\left(k\right)e^{-j(k_{n}\left(\omega \right)\lambda /8-\pi /2)}\times sin\left(\frac{k_{n}\left(\omega \right)\lambda +k\lambda }{8}\right)\;.$$

While the maximum locus lies at $k=-k_{n}\left(\omega \right)+4\pi /\lambda$, the minimum occurs at $k=-k_{n}\left(\omega \right)$. This leads to one obtaining the destructive interference at all frequencies for the wave mode *n*. This can be seen in Figure 5, where a complete overlap between the minimum locus (represented by the orange curve) and the dispersion curve (illustrated as the black arced line) of SH1 occurs on the left side. In contrast, on the right side, the maximum locus (shown as a blue curve) and the dispersion curve of SH1 do not overlap; instead, they intersect at the centre frequency. This indicates that the generated waves undergo a perfect destructive interference in the backward direction, but they do not form a perfect constructive interference in the forward direction.

The time-dependent excitation methods are expanded in [42] to completely suppress an unwanted wave mode in an undesired direction and generate distinct wave modes that simultaneously propagate in predefined directions.

The signal excitation methods require one to calculate the centre frequency of the base signal, $g_{0}\left(t\right)$, from the dispersive curve of the desired wave mode, while the nominal wavelength of the transducer is required for the time-delay based methods. Afterwards, the time delay is applied to the signal in the time domain to obtain the excitation signals $g_{1}$ and $g_{2}$. For the synthetic propagation-based methods (SP and SPI), one must determine the centre frequency using the dispersion curve and then apply the Fourier transform to the base signal before computing the excitation signals. These are computed in the frequency domain before applying an inverse Fourier transform to the excitation signals to determine the final excitation signal. It is worth observing that the SP and SPI methods are equivalent to the TD and TDI methods used for non-dispersive waves, respectively. These computations are crucial and aim to focus energy in a specific direction to achieve the maximum energy concentration possible in that desired direction. However, side lobes can appear alongside the main lobe due to physical and practical limitations in the wave generation process.

To evaluate the efficacy of unidirectional generation, the so-called “*unidirectionality*”, one can calculate the forward-to-backward wave amplitude ratio (FBWR). It is represented by the ratio of the forward-generated wave’s peak-to-peak amplitude to the backward-generated wave’s peak-to-peak amplitude in dB. Table 1 displays the calculated forward-to-backward ratio from [103,104,108]. The comparison is performed for SH modes SH0 and SH1 based on the signal excitation methods mentioned in the previous sections. While the authors of [103] generated SH0 and SH1 and showed results for TD, TDI, SP, and SPI, the authors in [104,108] generated only the fundamental mode SH0 and calculated the forward-to-backward wave ratio using TDLBPT and TD.

### 4.4. Side-Lobe Suppression for Unidirectional UGWs

**Figure 6:**
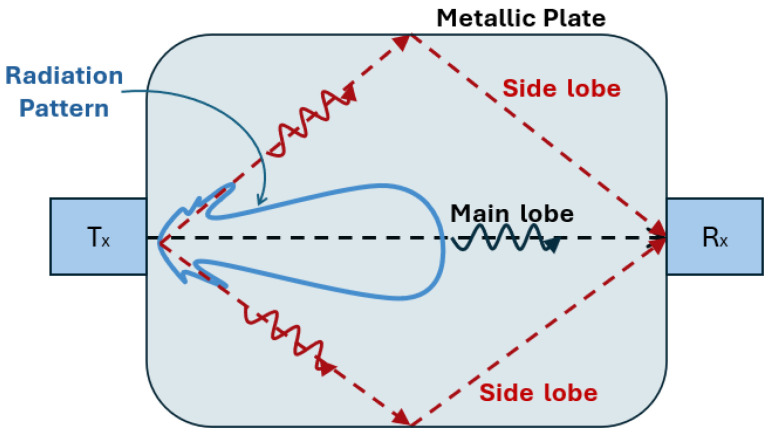
A metallic plate-like structure where an ultrasonic transducer with a transmitter, $T_{x}$, propagates waves and non-negligible side lobes appear. The waves generated by the side lobes in the transducer’s radiation pattern are reflected at the plate’s edge and reach the receiving transducer, $R_{x}$, as do those of the required main lobe, complicating signal interpretation. Redrawn based on [129].

The concepts of main lobes and side lobes are critical for understanding a wave’s unidirectional energy propagation and ensuring measurement accuracy. The main lobes are the primary path, where most wave energy is concentrated and then propagates in a specified direction. In unidirectional UGWs, this is the propagation path, in which one aims to maximise wave energy. Side lobes are secondary propagation paths, where some wave energy propagates in directions other than the intended main-lobe direction [105,128]. These are generally undesirable, because even with unidirectional guided waves they can cause interference, reduce the clarity of measurements, and lead to false indications [129,130]. Figure 6 illustrates the main side lobes in a metallic plate, in which the side-lobe waves can reflect from the edges towards the receiver, $R_{x}$, causing complications in interpreting the received waves.

Novel methods have been proposed in the literature to reduce side-lobe effects and increase directivity. One can develop multitrack transducer configurations with arrays of both phases distributed among two or three tracks and more minor, fewer, or even no gaps [131,132]. Others have also tried varying the magnets’ dimensions in the PPM array by following different shapes [129,130,133] or even decreasing the amplitude of the backward-travelling side lobes by increasing the number of magnet rows or reducing the lateral gap between rows [134].

**Figure 7:**
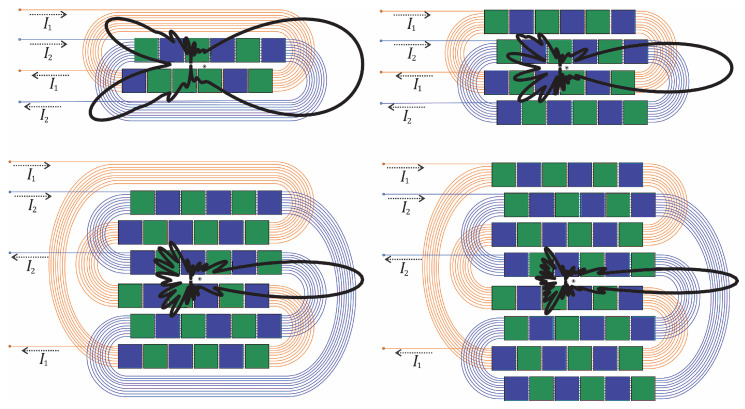
Side-shifted EMATs seen when increasing the number of PPM rows and expanding the coils from one row (**top-left**) to four rows in each array (**bottom-right**). The green and the blue square blocks are the magnets’ north and south poles, respectively. Redrawn based on [135].

A side-shifted periodic permanent magnet (PPM) EMAT with two independent PPM arrays was proposed in [135], which imposes a side shift to adapt PPM arrays and racetrack coils. Therefore, the wavefronts produced by the individual array are side-shifted, and the wavefields produced by each array mutually cancel only at the centreline of the array (*x*-axis), where they have the same amplitude. The resultant side lobes are lessened due to the rise in the number of rows and the reduction in side-shift separation; Figure 7 illustrates the proposed principle. Increasing the number of rows for each PPM reduces the backward-travelling side lobes because it lowers the relative misalignment between the individual wavefronts generated by each individual PPM array [135]. The proposed design, found in [135], represents an improvement compared to previously published designs. The authors managed to reduce the magnitude of the side lobes from −8 dB to −16 dB with four PPM rows and a concise, evaluated lateral separation of 2 mm. With three rows of PPM arrays, the side-lobe magnitude decreases to −13 dB due to a smaller device width. They also reported that utilising only two rows of PPMs decreases the side-lobe amplitude by 2 dB by reducing the lateral separation from 3 mm to 2 mm [135]. The lateral separation was further reduced to 1 mm in [136] by adopting flexible printed circuit board racetrack coils.

### 4.5. Analytical and Numerical Modelling of Unidirectional UGWs

Xie et al. [130] have proposed an EMAT which consists of double variable-length meander line coils and one permanent magnet that can generate unidirectional Rayleigh waves and suppress side lobes. They developed the two transmitter coils using one four-layer flexible printed circuit board with a thickness of 130 μm for each. The results in [130] indicated that the wire step determines the suppressed amplitude of the side lobes and also increases the beam width of the main lobe. For example, a 58.92% suppression of the side-lobe amplitude results in a 20% increase in the beam width of the main lobe. Therefore, it is essential to find a suitable balance between the beam width of the main lobe and the suppression of the side lobe, depending on the EMAT’s specific application.

These computations and design techniques are crucial for focusing ultrasonic energy in a single direction; however, finite element analysis is useful for simulating wave propagation before any actual physical prototype is created. It permits one to simulate, visualise, and analyse how ultrasonic waves propagate through different materials and components, allowing for the efficient and cost-effective development of ultrasonic devices.

While analytical models help users gain fundamental insights into their research, they often require simplifications that may only partially capture real-world complexities, especially complex geometries or heterogeneous materials [36,40,72,77,109,130,137]. For instance, a line-source-based mode for unidirectional SH wave EMATs was proposed in [137]. Numerical modelling, such as a finite element analysis, provides more detailed simulations of wave propagation within complex structures. Both analytical and numerical models are essential for understanding and optimising unidirectional ultrasonic wave generation and reception systems [41,81,101,102,106].

The finite element method (FEM) may be integrated with other numerical methods or analytical analyses to provide optimum solutions, ensuring the reliability of the final solution [69,138]. Finite element tools, such as Abaqus [76,78], Comsol Multiphysics [43,139,140], and OnScale [104] have been used to visualise numerical calculations to evaluate the performance of the unidirectional ultrasonic wave generation and reception principle. In addition to modelling UGW propagation, FEM models can also incorporate electromagnetic and mechanical fields and transducer configurations [140,141].

To summarise, the optimal generation of unidirectional guided waves is challenging but critical for enhancing the efficiency and accuracy of NDT&E. Careful control of signal excitation and wave propagation characteristics focuses UGWs in a specific direction, thereby minimising side lobes, multi-mode phenomena, and interference from unwanted directions. Considering the dispersion relations of unidirectional UGWs and their specific applications, the abovementioned techniques and methodologies can generate different UGW modes.

## 5. Unidirectional Ultrasonic Transducers

One strategy used to develop a unidirectional ultrasonic transducer is to use two arrays of an ultrasonic source in the manner illustrated in Figure 4, so that ultrasonic waves can constructively interfere in the desired direction and destructively interfere in the opposite direction. Many transducers operating based on different principles can be designed to generate and receive unidirectional guided waves. Here, we will introduce the three most commonly utilised ultrasonic transducers for the generation and reception of unidirectional UGWs.

### 5.1. Unidirectional Magnetostrictive Transducers

Unidirectional magnetostrictive transducers use magnetostrictive materials bonded to a sample that act as the sensing element [142,143]. Magnetostrictive materials, such as Terfenol-D, FeCo alloys, and nickel, change shape or dimension when exposed to a magnetic field in a phenomenon known as magnetostriction [134,144,145]. The material expands or contracts based on the direction and strength of the applied bias and dynamic magnetic fields [25,26,27]. Ultrasonic vibration is first produced in the magnetostrictive strips and the generated ultrasonic wave will then travel through the bonding layer into the sample. Therefore, this type of transducer can be used in the nondestructive testing of both ferromagnetic [25,144] and nonferromagnetic materials [146,147,148].

**Figure 8:**
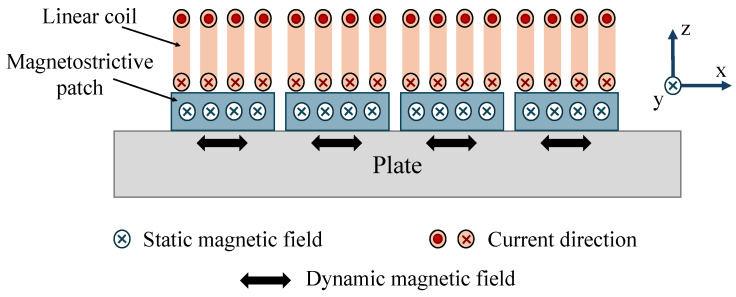
Configuration of a unidirectional wideband SH guided wave phased-array magnetostrictive patch transducer producing both a static magnetic field, coming from the magnetostrictive patches, and a dynamic magnetic field, generated by the alternating current flowing through the coils. The static magnetic field is perpendicular to the dynamic magnetic field. Redrawn based on [27].

A wideband SH guided wave phased-array magnetostrictive patch transducer (MPT), shown in Figure 8, was proposed by [27] to generate unidirectional SH waves. The authors have used a narrow magnetostrictive patch and a linear coil for each array element to increase the bandwidth of each single element. Then, by controlling each array element’s excitation delay and initial phase separately, they managed to control the directivity of the SH guided waves in the forward or backward direction and change the wavelength without replacing the coil.

A unidirectional magnetostrictive transducer has been proposed based on offsetting the permanent magnet to one side of the magnetostrictive strip in [149], in which the offset splits the magnetostrictive strip into two regions with opposite polarisations. This causes the strains produced in each region to be in opposite directions to each other; as a result, this will realise the unidirectional generation of a guided wave.

### 5.2. Unidirectional Piezoelectric Transducers

When a piezoelectric material is subjected to mechanical stress, it generates an electric charge across its surfaces. This effect is used in sensors to convert mechanical vibrations or pressure into electrical signals. When an electric field is applied to a piezoelectric material, it undergoes mechanical deformation. This effect is used in actuators and ultrasonic transducers to generate mechanical waves from electrical signals [101,150,151,152,153].

Standard piezoelectric transducer designs are developed using specific materials that have a piezoelectric effect, such as PZT [101,151], quartz [154], and LiNbO3 [46,155], as a metal substrate to provide the phase gradient required for unidirectional beam deflection without the need for a time delay during excitation and reception [151].

Realising unidirectional propagation using a piezoelectric transducer typically involves using two bidirectional wave sources that are in the same position. This is demonstrated in [150] with a symmetrical mounted transducer configuration, in which two different bidirectional wave transducers are symmetrically mounted on the upper and lower surfaces of the plate. Although the double-side arrangement is feasible in a laboratory setting, it becomes a challenge in real-world applications where accessing both surfaces can be difficult. To overcome this, the authors proposed a single-sided arrangement that allows same-position excitation. In this setup, the bidirectional piezoelectric transducers, face-shear and thickness-shear, are arranged in the direction perpendicular to wave propagation, allowing unidirectional excitation without a frequency-dependent time delay [150].

**Figure 9:**
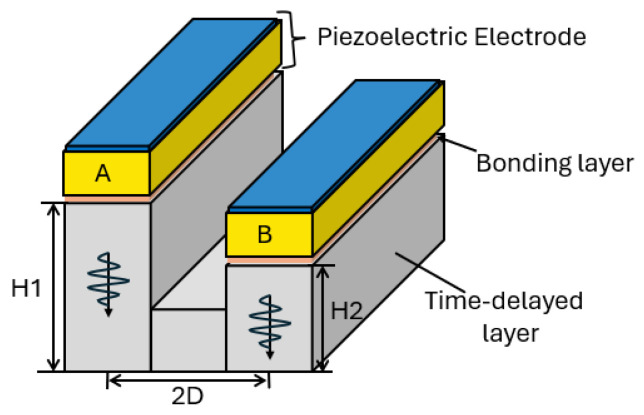
Schematic diagram of a time-delayed layer-based piezoelectric transducer. The transducer consists of time-delayed layers (H1 and H2) of different heights and two rectangular thickness-shear (d15)-mode piezoelectric wafers (A and B). D represents half the lateral spacing between the two line force sources. Redrawn based on [108].

A radar transducer for unidirectionally emitting and directing SH guided waves that is based on a time-delayed layer-based piezoelectric transducer was proposed in [109]. To obey the unidirectionallity principle, the authors added time-delay layers with different heights to each ultrasonic source to add longer delays to them, as seen in Figure 9. Then, they placed the transducer in a circular arrangement that contained multiple rectangular piezoelectric wafers and a circular metal substrate. The metal substrate had a ring base and several rectangular strips with two distinct heights and was based on the time-delay layer method. The rectangular strips were placed symmetrically on the ring base. The shorter rectangular strips were positioned outside the ring base, and the taller ones were placed on the inner side. Then, each of the polarised piezoelectric wafers in the length direction were positioned atop each rectangular strip, and the polarisation directions of the taller and shorter strips were opposite to each other, with the electrodes added on the upper and lower sides of the piezoelectric wafers. Using this configuration, an opposing thickness-shear wave (d15) was generated on the surfaces of two rectangular strips when applying the driving signal to the piezoelectric wafers simultaneously because of their opposite polarisation. As a result, two shear waves were excited within the strips, with a phase difference of π due to the height dissimilarity between the two rectangular strips (H1 and H2). The generated shear waves have approximately the same amplitudes when neglecting the bit of energy loss in the taller rectangular strips [109]. Consequently, and due to the intrinsic π phase difference, constructive and destructive interference emerged on the right- and left-hand sides, respectively.

Compared to EMATs (to be elaborated on in the next section), piezoelectric transducers are more effective in energy conversion and more accessible for manipulation. As a result of their high energy conversion efficiency, the phase-controlled pairs of antiparallel piezoelectric strips are expected to excite stronger unidirectional waves; however, piezoelectric transducers require a coupling agent layer, which plays a critical role in ensuring the efficient transmission of ultrasonic waves between the transducer and the material under inspection. Piezoelectric transducers rely on precise mechanical contact with the material to effectively generate or receive ultrasonic waves. A significant portion of the wave energy can be reflected back without proper coupling, resulting in signal loss or distortion. Using a coupling agent also limits the applications of piezoelectric transducers when samples are under elevated temperatures or in motion [108,156]. Alternatively, dry-coupled [157,158,159] and air-coupled piezoelectric transducers [160,161] have been developed as an alternative to those that use a liquid coupling agent.

### 5.3. Unidirectional Electromagnetic Acoustic Transducers (EMATs)

Unlike piezoelectric transducers, which require direct contact with the material, EMATs operate through electromagnetic fields, making them highly effective for non-contact and non-invasive testing. This property makes unidirectional EMATs particularly practical in industrial applications [69,104,130].

EMATs generate ultrasonic waves using electromagnetic induction principles. When a coil in the EMAT carries an alternating current, it generates a time-varying magnetic field. This magnetic field induces eddy currents on the surface of a conductive material; the interaction between these eddy currents and a static magnetic field (usually provided by a permanent magnet in the EMAT) results in a Lorentz force, which causes the material to vibrate locally, creating mechanical vibrations that propagate as ultrasonic waves [41,115].

**Figure 10:**
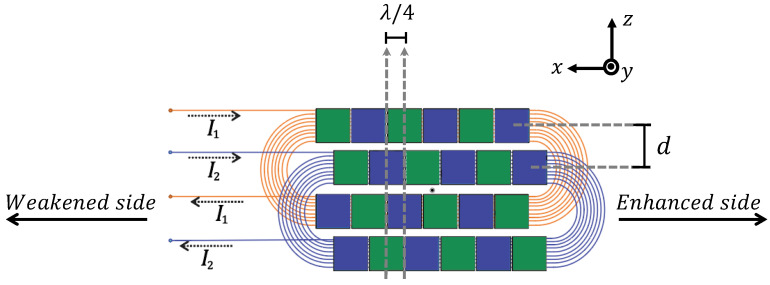
This diagram describes the operation principle of a side-shifted unidirectional dual PPM EMAT. The magnets’ north and south poles are represented by square blocks (green and blue), while blue and orange wires describe the two racetrack coils. The injected currents are, respectively, represented by $I_{1}$ and $I_{2}$, which are excited by a delay of 90^∘^. The enhanced and the weakened sides are shown on the right- and left-hand sides of the EMAT, respectively. The two coil sets are side-shifted by a distance *d* and longitudinally shifted by a quarter-wavelength (λ/4). Redrawn based on [44].

A side-shifted unidirectional PPM EMAT design was proposed in [44] for unidirectional generation. Figure 10 demonstrates the proposed EMAT, in which green and blue blocks represent the magnets’ poles, and the two racetrack coils are defined by blue and orange wires. The currents, $I_{1}$ and $I_{2}$, represented by dashed lines, are injected into coils 1 and 2, respectively. $I_{2}$ is 90^∘^ delayed compared to $I_{1}$. Moreover, the array and coil sets are side-shifted by a distance *d* and longitudinally shifted by a quarter-wavelength. The generated waves constructively interfere with each other on the right-hand side and destructively interfere on the left-hand side, even though their wavefronts are slightly side-shifted. An alternative design, without side-shift but using a dual linear-coil PPM, was proposed in [104].

Although a permanent magnet is an essential component in the EMATs, one can design a compact coil-only EMAT structure without bulky magnets or individual electromagnets for unidirectional transmission and the reception of Lamb waves in steel plates [45]. EMATs work well with conductive materials, particularly metals, due to the interaction between their magnetic field and the eddy currents in the metal, which generates the ultrasound. However, they are limited by their relatively high power consumption and the complexity of their setup. They are also less efficient at very high frequencies compared to piezoelectric transducers [162].

A meander-line dual-coil electromagnetic acoustic transducer (EMAT) consisting of a permanent magnet and two identical meander-line coils was proposed in [139]. The permanent magnet generates a magnetic field perpendicular to the specimen’s surface. The distance between the two coils is one-quarter of the wavelength λ/4. Both coils have the same parameters, with the spacing between adjacent wires in each coil set at half the wavelength λ/2. Two high-power burst currents supply power to the coils, each with the same amplitude but a 90^∘^ phase shift.

EMATs operate using the principle of electromagnetic induction to generate and detect ultrasonic waves in conductive materials. However, precise alignment of the EMAT’s components (magnets and coils) is essential to ensure accurate wave generation, mode selectivity, and optimal signal detection [107]. This is especially relevant for PPM-like unidirectional EMATs because they are composed of many magnets. When their alignment is performed incorrectly, even if slightly misaligned, one array might act on the other coil, generating undesirable out-of-phase Lorentz forces in the medium and restricting the transducer’s ability to generate unidirectional waves [107].

**Figure 11:**
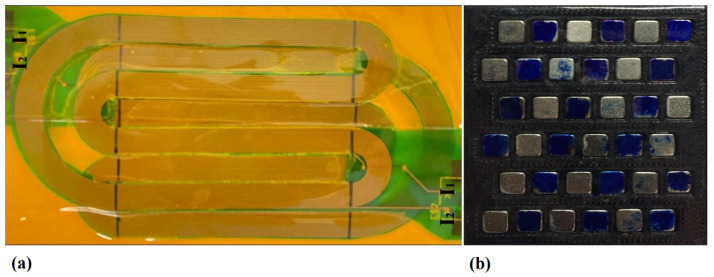
An example photograph of the multiple-row side-shifted PPM EMAT devised in [135] with the PCB dual-coil revised in [136], which includes (**a**) two coils carrying the currents $I_{1}$ and $I_{2}$ and (**b**) the rows of a PPM array.

Figure 11 shows an example photograph of the multiple-row side-shifted PPM EMAT devised in [135] with the printed circuit board (PCB) dual-coil revised in [136]. This transducer consists of two coils that are designed on a PCB, with two current connections, $I_{1}$ and $I_{2}$, that carry excitation signals.

In general, unidirectional ultrasonic transducers have their own unique strengths and challenges based on their operational principles and design. Piezoelectric transducers offer high-frequency operation and precision, making them suitable for applications that require detailed imaging or specific wave modes. EMATs, with their non-contact operation, provide a sense of safety in dangerous industrial settings. Magnetostrictive transducers can be a good choice for large-scale structures with their robustness and long-range capability. The selection of a transducer should be based on its specific application, the environmental conditions, and the required wave mode and frequency.

## 6. Applications of Unidirectional UGWs

Ultrasonic guided waves are a powerful tool for non-invasive inspection, monitoring, and characterisation in various industries. Their ability to propagate over long distances and their sensitivity to defects and material properties make them invaluable in ensuring the safety, efficiency, and reliability of structures and components. This technology’s continued development and application will likely expand its use within new and emerging fields [27,41,106,163,164]. Moreover, it is an effective tool for thickness measurements [37,165], especially in situations where access to only one side of a structure is available or when long-range inspection is required [37,101,124,140,163,166,167,168]. They can also be used in SHM systems to continuously monitor the health of large structures such as bridges, skyscrapers, and tunnels. By propagating along the structure’s length, these waves can detect cracks, stress, and other signs of deterioration over time [48,169]. In modern construction and aerospace, composite materials are increasingly used due to their high strength-to-weight ratio [4,27,76,101,114,150]. However, for the same aforementioned applications, unidirectional guided waves can provide even more advantages than guided waves because they simplify signal interpretation, as the waves can be received on one side and skipped on the other side, and also focus the energy into one direction, allowing longer distances along the structure to be investigated [33,34,37,38,39,48,76,102,168,170].

Researchers began outlining the applications and the influence of geometrical features on guided wave propagation, such as the effect of bends and step changes in the thickness of plates and pipelines when using long-range unidirectional guided waves [34,39], as far back as 1997. Cawley et al. [38] explored the use of ultrasonic guided wave screening on thousands of metres of pipework in petrochemical plants to detect and prevent unacceptable levels of corrosion. They proposed using two transducer rings to generate torsional and longitudinal unidirectional UGWs within the pipe wall. This method is advantageous as it allows for excitation from a single location on the pipe, enabling the unidirectional guided waves to propagate over tens of metres and return ultrasonic echoes without mixing with echoes reflected from the opposite direction. These echoes can indicate the presence of corrosion or other features within the pipe. Guided Ultrasonics Ltd. has commercialised this technique, developing products such as the Wavemaker Pipe Screening System [38], which inspects extensive lengths of pipelines from a single position.

In 2017, Breon and his colleagues [33] patented a method for directing guided waves along a pipeline in a specific direction, either backwards or forwards, depending on the area of interest. They utilised two transducer rings and controlled the current applied to each transducer by introducing an appropriate time delay. They also proposed a scanning system that displays the amplitude of the reflected wave energy in relation to the distance from the transducer array’s position. This innovative system can present reflected energy wave information in various graphical formats [33]. In 2019, Niu et al. [76] conducted experimental evaluations on a steel pipe measuring 11.6 m in length and 8 inches in diameter, with a defect of a size that represented 2% of its cross-sectional area (CSA) and that was located 3 m from the first ring. They utilised two and three rings of 24 equally distributed piezoelectric transducers for the experiments. The experimental setup involved a 10-cycle Gaussian-windowed pulse with a centre frequency of 35 kHz. The results demonstrated effective unidirectional excitation of the torsional wave mode T(0, 1), which enabled the detection of the 2% CSA defect. Additionally, the reflection ratios for the wall loss defects and the back-to-front ratios were measured: they were 1.63% and 3.67% for the two-ring setup and 1.62% and 7.69% for the three-ring setup, respectively [76]. The findings indicated that unidirectional excitation increased detection accuracy compared to traditional guided waves. Additionally, the performance of this system was further enhanced by the inclusion of an extra ring of transducers. However, it is essential to carefully adjust the setup when adding more rings to maintain the unidirectionality of the guided waves, as was further investigated in [77,78] in addition to the relationship between defect type, dimension, and transducer arrangement.

**Figure 12:**
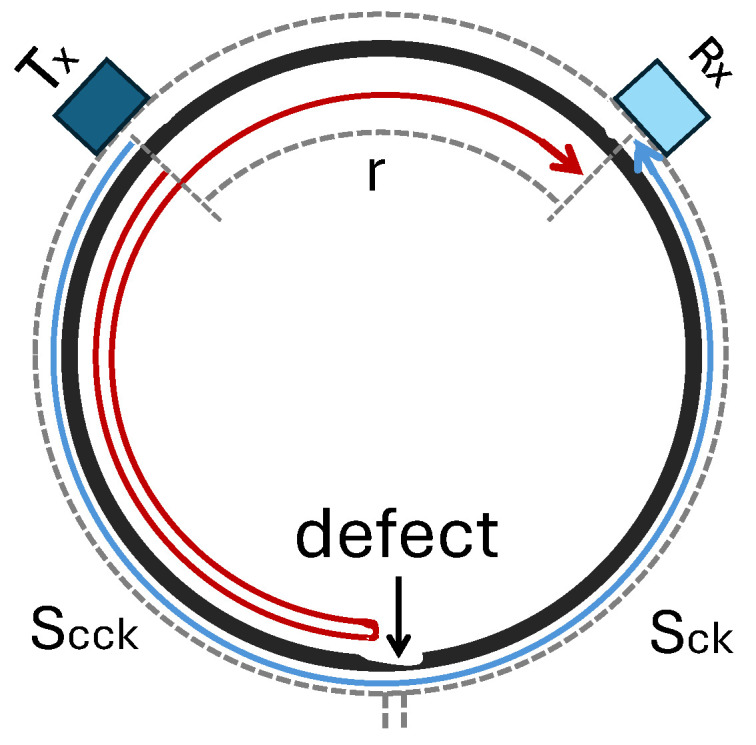
Schematic of wave propagation through a defect in one direction and reflected from the defect in the contrasting direction. $T_{x}$ represents the transmitter, while $R_{x}$ denotes the receiver. The blue and red arrows illustrate the passing and reflected waves. Redrawn based on [102].

Understanding the impact of defects on signal reception is crucial in wave propagation in a medium. Any defect changes the signal received at the receiver compared to a non-defective structure due to wave scattering or discontinuities. The travelling waves might experience partial reflection, forming a new wavefront that propagates backwards, and partial transmission through the defect in the forward direction, which usually delivers a lower amplitude than a direct wave with no defect [102,170]. For circumferential guided waves in pipes, as a closed-loop propagation path, bidirectional generation can complicate signal interpretation depending on the defect’s position [102,106]. The reflected signal received from a defect can blend with, or be masked by, the signal related to the direct wave propagating in the opposite direction. In this case, unidirectional guided waves are highly advantageous [102]. Figure 12 schematically illustrates the waves propagating in circumferential, where the waves that propagate through the defect and are reflected from the defect are represented by blue and red arrows, respectively. We assume the distance between the transmitter, $T_{x}$, and receiver, $R_{x}$, is *r*, and the defect is located at $s_{ckw}$ clockwise and $s_{cckw}$ counter-clockwise from the transmitter, with *p* as the pipe circumference. One can calculate the time at which the waves passing through the defect and the reflected waves are received at the receiver, $R_{x}$. When the times-of-arrival for the passing and reflected waves are equal, both waves arrive at the receiver simultaneously. This means the reflected wave from the defect can be masked by the generated wave, which can happen, for instance, when the angular position of the defect is diametrically opposite the transmitter [102], thus hindering signal interpretation. But unidirectional transmission avoids this, allowing for a clearer received signal, as illustrated in [102]. Moreover, combining unidirectional generation and reception further enhances interpretability, avoiding signal masking even if the transmitter and receiver positions are the same, as in a pulse-echo operation, as shown in [41].

A thickness measurement technique based on a unidirectional EMAT was proposed in [37]. The authors developed an experimental setup with a dual PPM EMAT to generate unidirectional SH wave modes predominantly in the forward direction. One conventional SH-wave EMAT receiver was positioned on the enhanced side of the unidirectional transmitter and another receiver was positioned on the weakened side. Then, the maximum forward-to-backward wave ratio (FBWR), defined as the ratio of the forward waves’ peak-to-peak amplitude to the backward waves’ peak-to-peak amplitude, was studied theoretically and experimentally. The authors established the mathematical link between the FBWR and the plate thickness and found that the FBWR is very sensitive to the thickness of the plate. Unlike conventional thickness measurement methods [168], which typically study the amplitude of ultrasonic guided waves on only one side of the transmitter, the method proposed in [37] took advantage of the information from both the enhanced and the weakened sides of the ultrasonic waves. The authors conducted experimental evaluations of their method using aluminium plates. They measured the plates’ thicknesses with an accuracy yielding an error of less than 0.8% [37]. This represents an error approximately three times lower and a quality factor exceeding ten times that of conventional methods. Ref. [37] appears to be the first reported research on measuring plate thickness by analysing both the enhanced and weakened sides of unidirectional guided waves at the same time, revealing a promising new application for the unidirectional UGW technique.

Unidirectional guided waves are essential for NDT&E and SHM because they enablw efficient, long-range inspection with minimal energy loss and high measurement accuracy. Their ability to make ultrasonic waves propagate in a specific direction improves their detection sensitivity, reduces unwanted signal interference, and allows for more accurate defect identification in materials and structures, which is vital for detecting cracks, corrosion, and other forms of structural degradation in various applications.

## 7. Conclusions and Future Directions

Unidirectional ultrasonic guided waves, a useful tool for nondestructive testing and evaluation (NDT&E) and structural health monitoring (SHM), offer enhanced selectivity, energy efficiency, and improved signal clarity. These waves are generated through carefully designed transducers such as piezoelectric, electromagnetic acoustic, and magnetostrictive transducers, which direct the energy into a single direction for propagation. This unidirectional propagation feature minimises ultrasonic wave interference, reflections, and mode conversion. It ersures cost-effectiveness, making unidirectional waves highly effective in environments with complex geometries or where precise defect detection is critical.

The various wave generation methods available, such as signal excitation and transducer design techniques, allow for greater control over wave directionality and mode excitation, leading to more focused energy propagation. However, challenges still exist, particularly in designing efficient transducers, optimising excitation signals, and dealing with complex wave interactions in intricate structures. Overall, unidirectional ultrasonic guided waves offer significant advantages over traditional guided wave techniques, particularly in applications requiring high-resolution detection and inspection within localised regions. With the recent advances in the optimal generation of unidirectional guided waves, the potential of this technique will be fully explored and this technique will chosen more frequently in practical applications.

Innovations in transducer technology, such as advanced materials, intelligent sensors, and adopting accelerators such as field-programmable gate arrays (FPGAs), can offer high-performance and reconfigurable digital signal processing capabilities to ultrasonic instruments [171,172,173,174]. Future transducers will aim to reduce side lobes, improve energy efficiency, and optimise the process of both the generation and reception of unidirectional ultrasonic guided waves. Integrating embedded systems into transducers could mean a significant jump forward in the applications of unidirectional ultrasonic guided waves. These advanced systems will offer enhanced sensitivity, real-time data processing, and higher reliability across various industries. As data from ultrasonic transducers become more complex, advanced machine learning and AI-based techniques will be essential for interpreting these data more accurately and efficiently [175,176,177,178,179]. Recent studies indicate that machine learning could outperform traditional closed-form algorithmic compensation methods [180,181], making it a promising area for future research and, therefore, able to significantly advance the utilisation of unidirectional ultrasonic guided waves in NDT&E.

## Figures and Tables

**Table 1:** Showing the unidirectionality achieved using time-delay (TD), time-delay and inverse (TDI), synthetic propagated (SP), synthetic propagated and inverse (SPI), and time-delayed layer-based piezoelectric transducer (TDLBPT) excitation methods. The unidirectionally is represented by the forward-to-backward ratio (FBWR) value, in dB, for the non-dispersive SH0 and dispersive SH1 modes. For the TDLBPT method used to generate SH0, the authors in [108] used 210 kHz. In [103], the authors employed 258.3 kHz to generate SH0 and 577.7 kHz for SH1, while the authors in [104] used 124 kHz. The dashed cells mean there is no experimental result provided by the reference for this method.

Article Ref.	TD (dB)		TDI (dB)		SP (dB)		SPI (dB)		TDLBPT (dB)
	SH0	SH1	SH0	SH1	SH0	SH1	SH0	SH1	SH0
[103]	22.4	12.3	37.7	15.7	22.4	08.4	37.7	29.5	-
[108]	-	-	-	-	-	-	-	-	22
[104]	20	-	-	-	-	-	-	-	-

## Data Availability

Dataset available upon request from the authors.

## Abbreviations

The following abbreviations are used in this manuscript:

SH	Shear Horizontal
SV	Shear Vertical
NDT	Nondestructive Testing
NDT&E	Nondestructive Testing and Evaluation
EMAT	Electromagnetic Acoustic Transducer
MPT	Magnetostrictive Patch Transducer
FEM	Finite Element Method
UGW	Ultrasonic Guided Wave
PPM	Periodic Permanent Magnet

PCB	Printed Circuit Board
CSA	Cross-Sectional Area
TD	Time-Delayed
TDI	Time-Delayed and Inverse
SP	Synthetic Propagated
SPI	Synthetic Propagated and Inverse
SHM	Structural Health Monitoring
FBWR	Forward-to-Backward Ratio
TDLBPT	Time-Delayed Layer-Based Piezoelectric Transducer
FPGAs	Field-Programmable Gate Arrays
